# Tumor microenvironment: driving forces and potential therapeutic targets for breast cancer metastasis

**DOI:** 10.1186/s40880-017-0202-y

**Published:** 2017-03-29

**Authors:** Hong-Yan Xie, Zhi-Min Shao, Da-Qiang Li

**Affiliations:** 1 Shanghai Cancer Center and Institutes of Biomedical Sciences, Shanghai Medical College, Fudan University, Shanghai, 200032 P. R. China; 2 Cancer Institute, Shanghai Medical College, Fudan University, Shanghai, 200032 P. R. China; 3 Department of Oncology, Shanghai Medical College, Fudan University, Shanghai, 200032 P. R. China; 4 Key Laboratory of Breast Cancer in Shanghai, Shanghai Medical College, Fudan University, Shanghai, 200032 P. R. China; 5 Department of Breast Surgery, Shanghai Medical College, Fudan University, Shanghai, 200032 P. R. China

**Keywords:** Breast cancer, Invasion and metastasis, Tumor microenvironment

## Abstract

Distant metastasis to specific target organs is responsible for over 90% of breast cancer-related deaths, but the underlying molecular mechanism is unclear. Mounting evidence suggests that the interplay between breast cancer cells and the target organ microenvironment is the key determinant of organ-specific metastasis of this lethal disease. Here, we highlight new findings and concepts concerning the emerging role of the tumor microenvironment in breast cancer metastasis; we also discuss potential therapeutic intervention strategies aimed at targeting components of the tumor microenvironment.

## Background

In terms of its molecular characteristics and clinical phenotypes, breast cancer is a highly heterogeneous malignancy. Like other solid tumor cells, breast cancer cells tend to metastasize to distant organs, such as the lung [1, 2], liver [2], bone [3], and brain [4], through local invasion and lymphatic and vascular dissemination. Metastasis is a continuous and multi-step biological process, in which a subpopulation of cancer cells with highly invasive and metastatic potential depart from their original locations, degrade the extracellular matrix (ECM), intravasate into the blood or lymphatic vessels, survive in the circulation, and extravasate to and colonize new terrain in the target organs [5, 6]. Although it is generally believed that breast cancer metastasis is a manifestation of late-stage and advanced disease, emerging evidence suggests that breast cancer cells can spread systemically at an early disease stage regardless of primary tumor size [7].

Distant metastasis attributes to approximately 90% of breast cancer-related deaths; as such, its underlying mechanisms are of intense interest and have been investigated extensively. Recently, several laboratories screened and identified a batch of breast cancer metastasis-related genes using well-established animal models of metastatic breast cancer in combination with high-throughput genomic analysis (Table 1). For example, it has been shown that secreted protein acid and rich in cysteine (SPARC), vascular cell adhesion molecule 1 (VCAM1), matrix metalloproteinase 2 (MMP2), and interleukin-13 receptor subunit α2 (IL-13Rα2) are involved in breast cancer lung metastasis [1], whereas interleukin-11 (IL-11) and connective tissue growth factor (CTGF) play a key role in breast cancer bone metastasis [3]. In addition, ST6 *N*-acetylgalactosaminide α-2,6-sialyltransferase 5 (ST6GALNAC5) mediates breast cancer brain metastasis [4]. Yet despite these remarkable advances, the precise molecular mechanisms behind breast cancer invasion and metastasis have not been fully delineated, therefore hampering the design of effective therapeutic strategies.

**Table 1:** Breast cancer metastasis-regulated genes

Target organ	Function(s) in breast cancer metastasis	Gene	Reference(s)
Lung	Promotion	*SPARC*	[1]
		*VCAM1*	[1]
		*MMP2*	[1]
		*IL*-*13Rα2*	[1]
		*α6β1/4*	[2]
		*Twist*	[17]
		*Par6*	[23]
		*BCAR4*	[37]
		*TNC*	[62]
		*CXCL12/CXCR4*	[73]
		*CCL2*	[74]
		*SERPINE2*	[79]
		*SLPI*	[79]
		*Coco*	[94]
	Suppression	*miR*-*126/miR*-126*	[35]
		*miR*-*708*	[36]
Bone	Promotion	*IL*-*11*	[3]
		*CTGF*	[3]
		*LOX*	[48]
		*CXCL12*	[53, 76]
		*14*-*3*-*3ζ*	[59]
		*JAG1*	[63]
		*VCAM1*	[95]
	Suppression	*miR*-*34a*	[32]
Liver	Promotion	*αVβ5*	[2]
		*PDK1*	[30]
Brain	Promotion	*ST6GALNAC5*	[4]
		*miR*-*19a*	[78]
Lymph node	Promotion	*CXCL12/CXCR4*	[73]
		*NRP2*	[81]

*SPARC*, secreted protein acid and rich in cysteine;* VCAM1*, vascular cell adhesion molecule 1;* MMP2*, matrix metallopeptidase 2;* IL-13Rα2*, interleukin-13 receptor subunit alpha-2;* Par6*, partitioning-defective protein 6;* BCAR4*, breast cancer anti-estrogen resistance 4;* TNC*, tenascin C;* CXCL12*, C–X–C motif chemokine ligand 12;* CXCR4*, C–X–C motif chemokine receptor 4;* CCL2*, C–C motif chemokine ligand 2;* SERPINE2*, serpin family E member 2;* SLPI*, secretory leukocyte peptidase inhibitor;* miR*, microRNA;* IL-11*, interleukin-11;* CTGF*, connective tissue growth factor;* LOX*, lysyl oxidase;* JAG1*, Jagged 1;* PDK1*, pyruvate dehydrogenase kinase 1;* ST6GALNAC5*, ST6* N*-acetylgalactosaminide alpha-2,6-sialyltransferase 5;* NRP2*, neuropilin 2

Since Paget proposed the well-known “seed and soil” theory of cancer metastasis in 1889 [8], accumulating evidence has shown that the interaction between breast cancer cells and the surrounding microenvironment is a determinant for breast cancer organ-specific metastasis [9]. The tumor microenvironment comprises stromal cells, bioactive molecules secreted by both tumor and stromal cells, ECM, and the lymphatic and vascular systems [10]. Stromal cells include immune cells, cancer-associated fibroblasts (CAFs), pericytes, and mesenchymal stem cells (Table 2). Secreted bioactive molecules by both tumor and stromal cells include growth factors, cytokines, chemokines, and exosomes [2, 10–13]. On the one hand, breast cancer cells educate the surrounding microenvironment to facilitate their invasion, survival, and growth in the manner of autocrine and paracrine. On the other hand, the tumor microenvironment adversely affects the invasive and metastatic potential of breast cancer cells. However, how these processes are regulated remains unclear.

**Table 2:** Stromal cells in breast cancer metastasis

Stromal cells	Function in breast cancer metastasis	Reference(s)
Immune cells		
Macrophages	Promotion	[40–44]
Neutrophils	Promotion/suppression	[45, 46, 96, 97]
T cells	Promotion/suppression	[44, 46, 49]
Natural killer cells	Suppression	[50, 51]
Cancer-associated fibroblasts	Promotion	[53, 76]
Pericytes	Suppression	[54, 55]
Mesenchymal stem cells	Promotion	[26, 56]

Here, we highlight new findings and emerging concepts regarding the role of the tumor microenvironment in breast cancer metastasis. Furthermore, we discuss the possibility of developing effective treatments by targeting components of the tumor microenvironment.

## Breast cancer cell features and breast cancer metastasis

### Epithelial-to-mesenchymal transition

Epithelial-to-mesenchymal transition (EMT) is a complex biological process in which epithelial cells are transited to a mesenchymal cell phenotype by genetic and epigenetic mechanisms [14–16]. The main feature of EMT is that epithelial cells lose phenotypes of cell polarity and cell–cell adhesion due to decreased or lost expression of cell adhesion molecules, such as E-cadherin (E-cad), while gaining a mesenchymal cell phenotype with highly migrative, invasive, and anti-apoptotic potential [10, 14–16]. The EMT program that is activated by several transcription factors, such as Snail, Slug, Twist, zinc finger E-box-binding homeobox 1/2 (ZEB1/2), and sex-determining region Y-box 4 (SOX4), plays a key role in multiple physiological processes, including embryonic development, chronic inflammation, and wound healing [10]. Interestingly, dysregulation of these transcription factors and related signaling pathways are implicated in breast cancer invasion and metastasis via activating the EMT program [17, 18].

Although many studies have demonstrated the critical roles of EMT in breast cancer metastasis, emerging evidence has shown that EMT may not be a prerequisite for triggering breast cancer metastasis. A case in point is paired related homeobox 1 (Prrx1), which is an EMT-inducing factor but inhibits breast cancer cell metastasis [19]. In support of these findings, Fischer et al. [20] found that only a small fraction of tumor cells undergo the EMT process in primary breast tumors, and lung metastatic cells still maintain epithelial cell characteristics. Moreover, suppression of EMT by overexpression of microRNA-200 (miR-200) does not affect the development of lung metastasis of breast cancer, but it does significantly enhance cellular response to chemotherapy [20]. These results suggest that EMT is not required for lung metastasis. Considering these emerging controversial data, the functional and mechanistic roles of EMT in breast cancer metastasis need to be further characterized.

### Cell polarity

Cell polarity is referred to as the asymmetry of cell morphology and the function and distribution of proteins. Cell polarity is tightly regulated by the polarity protein complex and has a significant role in cell proliferation, division, and migration and in tissue morphogenesis. The development of epithelial tumors is frequently accompanied by the loss of cell polarity, which leads to invasion and metastasis. For example, polarity protein partitioning defective 3 (Par3) is frequently down-regulated in breast cancer and can suppress mammary tumorigenesis and metastasis by inhibiting the expression of ECM-degrading enzyme matrix metalloproteinase 9 (MMP9) and the activation of the oncogenic JAK/STAT3 pathway [21, 22]. Moreover, Par3 loss is associated with aberrant activation of proto-oncogene *ErbB2*, a key regulator of the invasion and metastasis of breast cancer [22]. In addition, Par6-mediated cell polarity signaling pathways also play an important role in breast cancer metastasis [23]. In breast cancer xenograft models, inhibition of Par6 induces the formation of epithelial structure and inhibits breast cancer lung metastasis [23]. Furthermore, transcription factor ZEB1 can inhibit the expression of cell polarity gene lethal giant larvae homolog 2 (*Lgl2*), which is critical for maintenance of epithelial cell phenotype and is often down-regulated in breast cancer [24]. Thus, ZEB1-mediated down-regulation of Lgl2 may play a role in breast cancer metastasis [24].

### Breast cancer stem cells

In 2003, Al-Hajj et al. [25] successfully isolated breast cancer stem cells (BCSCs) from breast cancer specimens; since then, BCSCs have been considered an important factor for breast cancer metastasis and therapeutic resistance. Recently, Cuiffo et al. [26] showed that language development-associated gene forkhead box p2 (*FOXP2*) inhibits the amplification of BCSCs and the initiation and metastasis of breast cancer. Induction of miR-199a expression by mesenchymal stem cells (MSCs) increases the stemness of BCSCs through transcriptional repression of FOXP2 [26]. In addition, transcription factors Slug and Sox9 collaboratively maintain the stem cell state of BCSCs, and co-expression of these two transcription factors enhances the tumorigenic and metastatic ability of breast cancer [27]. Transcription co-activator with PDZ-binding motif (TAZ), a main downstream effector of the Hippo pathway, is highly expressed in BCSCs and poorly differentiated breast cancer cells, and is critical for the maintenance of self-renewal of BCSCs, as well as breast cancer growth, metastasis, and resistance to chemotherapy [28]. Therefore, further studies to elucidate the mechanisms of BCSCs in the progression and metastasis of breast cancer are crucial for the development of novel targeted therapeutic strategies.

### Tumor metabolism

According to the Warburg effect, most tumor cells increase aerobic glycolysis to reduce the levels of mitochondrial oxidative phosphorylation. To compensate for the lack of adenosine triphosphate (ATP), cancer cells increase glucose uptake and utilization. Recently, Fong et al. [29] found that breast cancer-secreted miR-122 suppresses glucose uptake of niche cells by down-regulating the glycolytic enzyme pyruvate kinase to enhance nutrient availability in the pre-metastatic niche and, thus, facilitate metastasis. In addition, Dupuy et al. [30] showed that different metabolic programs used by breast cancer cells determine the specificity of target organ of metastasis; they also showed that this process is controlled by pyruvate dehydrogenase kinase 1 (PDK1). In this context, unlike bone or lung metastatic cells, liver metastatic breast cancer cells exhibit a unique metabolic program, characterized by increased hypoxia inducible factor 1α (HIF1α) activity and enhanced expression of its target, PDK1 [30].

### Non-coding RNAs

Non-coding RNAs are the RNA molecules that are not translated into proteins. Of these, miRs are a group of evolutionarily conserved short (20–24 nucleotides) non-coding RNAs that are involved in post-transcriptional regulation of gene expression. Emerging evidence suggests that the abnormal expression of miRs can either promote or suppress breast cancer metastasis. miR-10b is highly expressed in metastatic breast cancer cells and promotes cell migration, invasion, and metastasis [31]. Ma et al. [31] have shown that Twist transcriptionally up-regulates miR-10b, which, in turn, suppresses the expression of the metastasis-suppressing gene homeobox D10 (*HOXD10*) and up-regulates the expression of the metastasis-promoting gene *Rho*.

Not surprisingly, some miRs have been shown to function in breast cancer as metastasis suppressors. For example, miR-34a suppresses osteoclast formation and bone metastasis by inhibiting the expression of osteoclastogenesis-inducing factor and transforming growth factor β (TGFβ)-induced factor 2 (TGIF2) [32]. Endogenous miR-126 is down-regulated in primary breast cancer tissues [33] and can suppress endothelial cell recruitment, angiogenesis, and metastatic formation by modulating the expression of insulin-like growth factor-binding protein 2 (IGFBP2), C-Mer tyrosine kinase (MERTK), and phosphatidylinositol transfer protein, cytoplasmic 1 (PITPNC1) [34]. In addition, miR-126/miR-126^*^ directly inhibits chemokine C–X–C motif ligand 12 (CXCL12) and indirectly suppresses chemokine C–C motif ligand 2 (CCL2) in a CXCL12-dependent manner, thereby blocking the recruitment of MSCs and inflammatory monocytes to the tumor microenvironment and reducing the lung metastatic potential of breast cancer [35]. In addition to miR-126, miR-335 is also down-regulated in primary breast tumors and suppresses breast cancer metastasis by targeting progenitor cell transcription factor SOX4 and extracellular matrix component tenascin C [33]. miR-708 is significantly down-regulated in metastatic triple-negative breast cancer and is epigenetically silenced by enhancer of zeste 2 polycomb repressive complex 2 subunit (EZH2), which is a core subunit of the polycomb repressive complex 2 (PRC2) [36]. Consistently, induction of miR-708 blocks lung metastasis of triple-negative breast cancer by, at least in part, inhibiting endoplasmic reticulum calcium-regulatory protein neuronatin [36].

Long non-coding RNA (lncRNA) is another type of non-coding RNA that is longer than 200 nucleotides. lncRNA was initially considered a byproduct of RNA polymerase II-mediated transcription without any biological functions. Recent studies showed that lncRNAs play important roles in the progression and metastasis of breast cancer [37, 38]. For example, the lncRNA breast cancer anti-estrogen resistance 4 (BCAR4) is highly up-regulated in advanced breast cancer patients and promotes breast cancer metastasis by activating a noncanonical hedgehog/glioma-associated oncogene family zinc finger 2 (GLI2) transcriptional program that promotes cell migration [37]. In contrast, nuclear factor kappa B (NF-κB)-interacting lncRNA (NKILA) has been shown to suppress breast cancer growth and metastasis induced by overactivation of NF-κB signaling which is caused by various inflammatory stimuli [38]. Consistently, breast cancer patients who have low expression of NKILA have a poor prognosis [38].

## Stromal cells and breast cancer metastasis

### Immune cells

In each step of the metastatic cascade, cancer cells are exposed to various types of immune cells, such as macrophages, lymphocytes, and monocytes, which can recognize them and regulate their growth and progression [39]. For example, clinical and experimental evidence has shown that macrophages play important roles in breast cancer growth, invasion, and metastasis [40, 41]. First, macrophages create an inflammatory microenvironment during the initiation phase to promote tumor growth [40, 41]. Second, macrophages stimulate angiogenesis, tumor cell invasion, and migration and suppress anti-tumor immunity as tumors progress to malignancies [40, 41]. Third, macrophages promote tumor cell extravasation, survival, and subsequent growth at the metastatic sites [40, 41]. Fourth, VCAM1 is highly expressed in breast cancer cells and recruits tumor-associated macrophages (TAMs) to the tumor microenvironment, triggering Akt activation in cancer cells and protecting cancer cells from cytokine-induced apoptosis [42]. Fifth, the interplay between macrophages and mesenchymal-like breast cancer cells is crucial for breast cancer metastasis [43]. In this context, mesenchymal-like breast cancer cells secrete granulocyte–macrophage colony-stimulating factor (GM-CSF) to promote the transformation of macrophages into TAMs. In turn, chemokine CCL18 secreted by TAMs induces mesenchymal-like breast cancer cells to activate the EMT program, thus creating a positive feedback loop between macrophages and mesenchymal-like breast cancer cells to promote invasion and metastasis of breast cancer cells [43]. Inhibition of GM-CSF or CCL18 significantly reduces breast cancer metastasis [43]. Finally, CD11 antigen-like family member B (CD11b)-positive, interferon gamma receptor 1 (Gr1)-negative, and cell surface glycoprotein F4/80-positive (CD11b^+^Gr1^−^F4/80^+^) tumor-associated macrophages trigger metastasis of breast cancer via activation of the epidermal growth factor receptor (EGFR) signaling pathway [44]. Together, these findings indicate that macrophages play a key role in breast cancer metastasis.

Neutrophils are the most common immune cells in the body, but their role in breast cancer metastasis remains controversial. Before metastatic breast cancer cells arrive in the lung tissue, neutrophils are gathered at the pre-metastatic site in the lung and produce leukotrienes, which recruit highly tumorigenic breast cancer cells to the lung and thereby promote the seeding and colonization of breast cancer cells [45]. Consistently, inhibition of leukotriene-producing enzyme arachidonate 5-lipoxygenase (Alox5) blocks neutrophil-mediated lung metastasis [45]. In addition, tumor-induced neutrophils promote the migration of breast cancer by inhibiting cytotoxic CD8^+^ T lymphocytes [46]. However, other studies showed that neutrophils can inhibit breast cancer metastasis by producing hydrogen peroxide and nitric oxide [47, 48].

T cells are the main component of lymphocytes with different subgroups, and their role in breast cancer metastasis is complicated. CD4-positive T lymphocytes promote lung metastasis of breast cancer by directly enhancing the function of tumor-associated CD11b^+^Gr1^−^F4/80^+^ macrophages [44]. Interleukin-17 (IL-17)-producing γδ T cells and neutrophils synergistically promote pulmonary and lymph node metastasis [46]. The underlying mechanism is that interleukin-1β (IL-1β) enhances the expression of IL-17 in γδ T cells, resulting in granulocyte colony-stimulating factor (G-CSF)-dependent expansion and polarization of neutrophils in mice with breast cancer. This kind of tumor-induced neutrophils suppress the function of cytotoxic CD8^+^ T lymphocytes, which limit the establishment of metastasis [46]. Additionally, tumor-infiltrating regulatory T cells promote breast cancer metastasis by stimulating the receptor activator of the nuclear factor kappa B ligand (RANKL) signaling pathway [49].

Natural killer (NK) cells are major effector cells of the innate immune system. NK cells are enabled to recognize and eliminate early malignant cells, thus preventing breast cancer tumorigenesis and metastasis [50]. Soluble MHC class I chain-related protein A (MICA) and MICB, two ligands of NK cell receptor D (NKG2D), can activate NK cells after binding NKG2D and have the function of immune surveillance. Interestingly, breast cancer stem cells decrease the expression of MICA and MICB, thus escaping from NK cell-mediated cytotoxicity and promoting metastasis [51].

### Cancer-associated fibroblasts and pericytes

Cancer-associated fibroblasts are the most important host cells in the tumor microenvironment. In breast cancer, CAFs play a central role in the tumor microenvironment since they promote angiogenesis, ECM remodeling, metabolic reprogramming, invasion and metastasis, and therapeutic resistance [52]. For example, CAF-derived CXCL12 and insulin-like growth factor 1 (IGF1) can promote the survival and outgrowth of highly invasive and metastatic triple-negative breast cancer cells in the bone marrow [53].

Pericytes are a kind of cells surrounding capillary and venous endothelial cells. Recent studies showed that pericytes in the microenvironment inhibit the metastatic potential of breast cancer cells [54, 55]. Depletion or pharmacological inhibition of pericytes is associated with increased hypoxia, EMT, and proto-oncogene *Met* activation [54]. Other studies showed that inhibition of the function of pericytes increases lung metastasis of hypoxic breast cancer cells by activating the angiopoietin-2 (ANG2) signaling pathway [55].

### Mesenchymal stem cells

Mesenchymal stem cells are the progenitors of stromal cells in the tumor microenvironment, contributing to the formation of tumor-associated stroma and metastasis [26, 56]. Recently, Karnoub et al. [56] showed that bone marrow-derived human MSCs can enhance the metastatic potential of human breast cancer cells. The underlying mechanism for these observations is that MSC-secreted chemokine CCL5 acts in a paracrine manner on breast cancer cells to enhance their motility, invasion, and metastasis [56]. In addition, Chaturvedi et al. [57] showed that MSCs were recruited to the primary breast cancer tissues to promote lymph node and lung metastases. Moreover, miR-126/miR-126^*^ reduces the lung metastatic potential of breast cancer by inhibiting the expression of CXCL12, thereby blocking the recruitment of MSCs to the tumor ECM [35]. These studies suggest that the interaction of breast cancer cells and MSCs in the microenvironment plays a key role in metastasis of breast cancer. Therefore, MSCs are expected to be a key target for the inhibition of breast cancer metastasis.

## Secreted bioactive molecules by tumor and stromal cells and breast cancer metastasis

### Cytokines

Transforming growth factor β is a multifunctional cytokine that plays a dual role in cancer development. TGFβ acts as a tumor suppressor in the initial stage of tumorigenesis but as a metastasis promoter in advanced cancers [58, 59]. TGFβ produced by breast cancer cells and stromal cells through an autocrine or paracrine manner promotes breast cancer metastasis via multiple pathways. For example, the TGFβ-Smad pathway induces the expression of interleukin-11 (IL-11), connective tissue growth factor (CTGF) [3], angiopoietin-like 4 (ANGPTL4) [60], and cut-like homeobox 1 (CUTL1) [61] to promote breast cancer invasion and metastasis. Furthermore, TGFβ forms a complex with mutant p53 and Smad to promote breast cancer cell migration, invasion, and metastasis by inhibiting tumor suppressor protein p63-mediated signaling pathways [58]. Consistently, lysine-specific demethylase 1 (LSD1) and tumor suppressor gene deleted in liver cancer 1 (DLC1) suppress breast cancer invasion and metastasis by inhibiting the TGFβ pathway [62, 63].

In addition to TGFβ, cytokine tumor necrosis factor α (TNFα) also induces breast cancer cell migration and invasion by enhancing the stability of Snail, which is a major transcription factor governing the EMT program [16, 64]. The underlying mechanism for this event is that TNFα induces the expression of COP9 signalosome 2 (CSN2) in a NF-κB-dependent manner. In turn, CSN2 inhibits the ubiquitination and degradation of Snail to increase its stability [64]. Interestingly, the release of TNFα from macrophages is regulated by serglycin, which is a proteoglycan that is highly expressed in [65] and constitutively secreted by breast cancer cells [66]. Emerging evidence suggests that elevated levels of serglycin can enhance breast cancer cell growth, migration, and invasion [65]. In a MMTV-PyMT-driven mouse model, genetic ablation of serglycin blocked lung metastasis of breast cancer [67]. In addition, serglycin can bind to cell surface adhesion molecule CD44 and mediates cell–cell interactions, cell adhesion, and cell migration in human cancers [68].

### Chemokines

Chemokines are a family of small cytokine-like molecules that control a wide variety of biological and pathologic processes, such as immune responses, viral infection, and cancer metastasis [69]. By binding to specific receptors on the membrane of the target cells, chemokines can recruit immune cells to the tumor microenvironment, thus regulating immune surveillance, angiogenesis, invasion, and metastasis [70]. To date, approximately 50 chemokines and 20 chemokine receptors have been identified. According to the spacing of their first two cysteine residues, chemokines can be divided into at least four families: C, CC, CXC, and CX3C [71]. CXC chemokines mainly act on neutrophils, whereas C, CC, and CX3C chemokines mainly regulate monocytes, macrophages, and lymphocytes [72].

Since Muller et al. [73] first reported in 2001 that the chemokine–chemokine receptor pathway was involved in the metastasis of breast cancer, the functional roles of chemokines and chemokine receptors in breast cancer metastasis have been extensively investigated (Table 3). For example, CCL2 produced by breast cancer cells and stromal cells recruits CC chemokine receptor 2 (CCR2)-positive inflammatory monocytes to the lungs; then these inflammatory monocytes secrete vascular endothelial growth factor to promote breast cancer cell extravasation and lung metastasis [74]. Thus, inhibition of CCL2–CCR2 signaling blocks lung metastasis of breast cancer [74]. In addition, TAMs produce CCL18; binding of CCL18 to its specific receptor PITPNM family member 3 (PITPNM3) facilitates breast cancer metastasis through activation of intracellular calcium signal [13]. Similarly, CXCL12 produced by CAFs mediates bone metastasis of triple-negative breast cancer [53]. In addition, human epidermal growth factor receptor 2 (HER2) enhances the expression of C–X–C motif chemokine receptor 4 (CXCR4), which is required for HER2-mediated invasion and metastasis of breast cancer [75]. Moreover, binding of bone marrow-derived CXCL12 to its receptor CXCR4 can activate Akt, thus promoting breast cancer bone metastasis [76].

**Table 3:** Breast cancer metastasis-associated chemokines and their receptors

Chemokine	Chemokine receptor	Reference(s)
CCL2	CCR2	[74]
CCL5	CCR5	[56]
CCL18	PITPNM3	[13]
CCL21	CCR7	[73]
CXCL1/2	CXCR2	[11]
CXCL10	CXCR3	[57]
CXCL12	CXCR4	[35, 73, 76]
CXCL8	CXCR1/2	[98]

*CCL* C–C motif chemokine ligand, *CCR* C–C motif chemokine receptor, *CXCL* C–X–C motif chemokine ligand, *CXCR* C–X–C motif chemokine receptor, *PITPNM3* phosphatidylinositol transfer protein membrane-associated 3

### Exosomes

Exosomes are small extracellular vesicles secreted to the extracellular environment by tumor cells and stromal cells and play key roles in cell–cell communications [77]. Recent studies showed that fibroblast-secreted exosomes promote the migration of breast cancer cells via Wnt-planar cell polarity (PCP) signaling [77]. Interestingly, tumor-derived exosomes determine organotropic metastasis in breast cancer [2]. In this context, exosomal integrins α6β4 and α6β1 are associated with lung metastasis, whereas exosomal integrin αvβ5 is linked to liver metastasis of breast cancer [2]. In addition, brain astrocyte-secreted exosomes carrying miR-19 regulate brain metastases of breast cancer by down-regulating the expression of tumor suppressor gene phosphatase and tensin homolog (*PTEN*) [78].

## The lymphatic and vascular system and breast cancer metastasis

As mentioned earlier, breast cancer metastasis requires that primary tumor cells possess the ability to intravasate into the blood or lymphatic vessels and extravasate into and colonize secondary sites [79]. Vascular mimicry contributes to not only vasculature of primary tumors but also metastasis to distant organs. Recently, Wagenblast et al. [79] showed that the secreted proteins serpin family E member 2 (SERPINE2) and secretory leukocyte peptidase inhibitor (SLPI) are primary regulators of vascular mimicry, and overexpression of SERPINE2 and SLPI is closely related to lung metastasis of breast cancer. In addition, vascular remodeling factors, including epiregulin, cyclooxygenase 2 (COX2), MMP1, and MMP2, can promote breast cancer lung metastasis through facilitating the release of tumor cells into the circulation system and breaching lung capillaries by circulating tumor cells [80]. Neuropilin 2 (NRP2) can modulate lymphangiogenesis, but its relationship with the metastasis of breast cancer is unclear. A recent study has shown that inhibition of NRP2 disrupts vascular endothelial growth factor C-induced lymphatic endothelial cell migration, reduces tumor lymphangiogenesis, and inhibits metastasis to sentinel lymph nodes and distant organs by delaying the departure of tumor cells from the primary tumor [81]. These findings indicate that NRP2 is an attractive target for modulating metastasis.

## Hypoxic microenvironment

Mammary tumor cells are always under a hypoxic microenvironment due to abnormal proliferation of cancer cells as well as dysregulation of vasculature structure and function. Hypoxia-inducible factor (HIF), composed of α and β subunits, is a master regulator of the hypoxic response. HIF-mediated adaption to hypoxia is mainly through transcriptional regulation of its downstream target genes that are involved cellular metabolism, angiogenesis, cell migration and invasion, autophagy, and cell death [47]. One of these downstream targets is lysine oxidase (LOX), which can stabilize ECM by oxidizing amino acid residues of collagen and elastin. Recent studies showed that multiple hypoxia-HIF signaling pathways promote breast cancer metastasis by inducing the expression of LOX [48, 82, 83]. LOX is highly expressed in hypoxic breast cancer cells and related to the invasive and metastatic potential of breast cancer by modulating focal adhesion kinase (FAK) activity and cell-ECM adhesion [83]. In addition, LOX secreted by hypoxic cells regulates the bone metastasis of estrogen receptor-negative breast cancer [48]. Moreover, LOX is important for the formation of the pre-metastasis microenvironment [82]. In this context, LOX is accumulated at the metastatic target organs and cross-linked with type IV collagen in basement membrane. Bone marrow-derived cells adhere to the cross-linked type IV collagen to promote the generation of MMP2, which, in turn, facilitates the degradation of collagen, thereby recruiting metastatic tumor cells to colonize the target organs [82].

Given the key role of HIF in breast cancer metastasis, upstream signals that regulate HIF could be involved in breast cancer metastasis. One case in point is SHARP1, also known as basic helix-loop-helix family member E41 or differentiated embryonic chondrocyte expressed gene 2, which suppresses the invasion and metastasis of triple-negative breast cancer by promoting the degradation of HIF1α via mediating its interaction with proteasome [84]. SHARP1 is also regulated by a metastasis suppressor gene, *p63* [84].

## Conclusions and perspectives

Breast cancer metastasis is the result of the interplay between tumor cells and the surrounding microenvironment, which is regulated by complex molecular networks. Although much progress has been made in understanding this process, effective therapeutic strategies against breast cancer metastasis are still lacking. One reason for this is the lack of suitable model systems that would enable us to fully reveal the underlying mechanisms.

Evidence is mounting that trophoblast invasion of the maternal endometrium during embryo implantation and cancer invasion of host tissues share striking similarities in numerous biological processes, such as cell migration, invasion, angiogenesis, and immune escape [85–88]. However, unlike unlimited tumor invasion and metastasis, embryo implantation is considered a natural model of successfully controlled tissue invasion [89, 90], which is strictly regulated through a delicate crosstalk between trophoblasts and the maternal endometrial microenvironment [87, 91–93]. Thus, unraveling the role of the maternal endometrial microenvironment in controlling trophoblast invasion during embryo implantation may provide new perspectives for understanding the molecular mechanisms of breast cancer metastasis and for developing novel therapeutic strategies for metastatic disease.
